# Factors influencing access to mental health services among adolescents and young people living with HIV in Zambia: a qualitative study

**DOI:** 10.21203/rs.3.rs-8559388/v1

**Published:** 2026-01-29

**Authors:** Carlos Muleya, Rosemary N. Likwa, Jacqueline J. Folotiya, Jeremia Banda, Bupe M. Kabamba, Lweendo Mapiki, Patricia Sakala, Caitlin Baumhart, Cassidy W. Claassen, Chikoloma Nakazwe, Naeem Dalal, Loyd Mulenga, Peter Chipimo

**Affiliations:** University of Zambia; University of Zambia; University of Zambia; University of Zambia; University of Lusaka; Cazacha Business Solutions Limited; Cazacha Business Solutions Limited; University of Maryland, Baltimore; University of Maryland, Baltimore; Zambia National Public Health Institute; Zambia National Public Health Institute; Ministry of Health; World Health Organization - Zambia

**Keywords:** Adolescent and young people living with HIV (AYPLHIV), Mental health access, Barriers and facilitators, Stigma and cultural beliefs, Peer support, Decentralization of health services, Mobile health (mHealth), Digital integration, Zambia, Qualitative study

## Abstract

**Background:**

AYPLHIV understand mental health but face socio-cultural, economic and technological barriers to care. This study suggests that decentralized services, stronger community and peer support and carefully designed mHealth tools–if youth-centered, confidential, affordable and integrated into HIV care–could help improve access and acceptability of mental-health support for young people living with HIV in Zambia and similar settings.

**Methods:**

AYPLHIV understand mental health but face socio-cultural, economic and technological barriers to care. This study suggests that decentralized services, stronger community and peer support and carefully designed mHealth tools–if youth-centered, confidential, affordable and integrated into HIV care–could help improve access and acceptability of mental-health support for young people living with HIV in Zambia and similar settings.

**Results:**

AYPLHIV understand mental health but face socio-cultural, economic and technological barriers to care. This study suggests that decentralized services, stronger community and peer support and carefully designed mHealth tools–if youth-centered, confidential, affordable and integrated into HIV care–could help improve access and acceptability of mental-health support for young people living with HIV in Zambia and similar settings.

**Conclusions:**

AYPLHIV understand mental health but face socio-cultural, economic and technological barriers to care. This study suggests that decentralized services, stronger community and peer support and carefully designed mHealth tools–if youth-centered, confidential, affordable and integrated into HIV care–could help improve access and acceptability of mental-health support for young people living with HIV in Zambia and similar settings.

## 1 Background

Adolescents and young people living with HIV (AYPLHIV) face an elevated risk of mental-health problems due to stigma, discrimination, and chronic disease stressors [1–3]. Globally, about one in three AYPLHIV experience depression or anxiety, with mental ill-health linked to poor adherence to antiretroviral therapy (ART), viral rebound, and suicidal ideation [4, 5]. Zambian studies indicate that approximately one in four adolescents living with HIV experience clinically significant depressive symptoms (25.3%) [36]. In a district-level study, Bwalya et al., used the Strengths and Difficulties Questionnaire (SDQ) to screen HIV-positive adolescents and found that more than 75% had emotional or behavioral difficulties [37].

The World Health Organization (WHO) identifies mental disorders as a leading cause of disability among adolescents, yet access to care in sub-Saharan Africa remains minimal [6]. In Zambia, approximately 1.5 million people live with HIV, and adolescents and young people (AYP) aged 15–24 years constitute 11% of this population [7]. With fewer than 10 psychiatrists serving a population of 18 million, most AYPLHIV rely on overstretched primary-health facilities that seldom offer structured psychosocial support [10]. Despite policy recognition, mental-health services (MHS) are highly centralized and under-resourced. The Mental Health Act (2019) provides a legislative framework for rights-based care, but implementation is constrained by insufficient funding, few trained specialists, and weak community integration [8, 9]. Socio-cultural beliefs, stigma, gender norms, economic and geographic inequalities further widen the care seeking and treatment gap [11, 12, 13].

Recent global evidence highlights the potential of digital and community-based innovations to address these gaps. Mobile-health (mHealth) platforms – ranging from SMS-based counseling to smartphone applications – offer confidential, low-cost, and scalable psychosocial support [14–17]. In Zambia, initiatives such as U-Report demonstrate that youth readily engage with digital tools for health information [18]. However, little is known about the cultural acceptability, and policy alignment of mHealth integration into mental-health delivery for AYPLHIV.

This study therefore explored AYPLHIV’s understanding of mental health, barriers and facilitators to accessing mental-health services, and the acceptability and policy alignment of integrating mHealth into mental-health delivery in Zambia.

## 2 Methods

### 2.1 Study Design

We used a cross-sectional qualitative study design guided by a phenomenological orientation – focusing on participants’ understanding of mental health, barriers and facilitators to access, and potential integration of mHealth innovations into existing service delivery systems. The analysis followed the Consolidated Criteria for Reporting Qualitative Research (COREQ) guidelines to ensure methodological transparency, rigor, and credibility [38].

### 2.2 Study Setting

Data was collected in two contrasting districts, Lusaka (urban) and Shibuyunji (rural), to represent rural-urban diversity in access to mental-health among AYPLHIV.

Lusaka District, the capital city, has the largest concentration of AYPLHIV in the country due to high HIV prevalence and population density. Based on national program data, an estimated 11,837 AYP aged 15–24 years are living with HIV in Lusaka alone. Applying Zambia’s documented prevalence of 25.3% experiencing clinically significant depressive symptoms among AYPLHIV and more than 75% screening positive for emotional or behavioral difficulties, this suggests that 2,995 AYPLHIV may be living with depression and up to 8,878 may be experiencing broader psychosocial challenges in Lusaka district. Lusaka district also hosts Chainama Hills Hospital, the country’s main psychiatric facility, making it a critical reference point for understanding gaps in centralized mental-health care.

Shibuyunji district, by contrast, is a sparsely populated rural district with limited health-care infrastructure. ART program shows that about 825 AYP are living with HIV in Shibuyunji district. Applying the same Zambia-specific prevalence rates, approximately 209 of these AYPLHIV may be experiencing depressive symptoms, and 619 may have emotional or behavioral difficulties–often without access to specialized care. Shibuyunji’s health system relies primarily on primary-care clinics staffed by nurses and lay counsellors, with no resident mental-health specialists.

Together, these settings allowed the study to capture urban–rural contrasts in mental-health service availability, sociocultural norms, digital access, and health-system capacity. They also represent two areas where AYPLHIV are likely to face markedly different pathways to mental-health support, making them ideal for exploring contextual determinants of access.

The districts were purposively selected due to proximity, while providing an opportunity to capture contextual variations in access, infrastructure, and cultural norms influencing youth mental-health service utilization.

### 2.3. Data Collection

We interviewed AYPLHIV through Focus Group Discussion (FGD) and In-Depth-Interviews (IDI), and policy makers using KIIs. Table 1 summarizes the respondent characteristics.

#### 2.3.1. Adolescents and Young People Living with HIV (AYPLHIV)

We administered four (4) FGDs and ten IDIs to AYPLHIV, with each FGD having with ten (10) participants. Two of the FGDs were sex disaggregated while the other two each had a combination of both male and female participants. One of the FGDs was conducted in a Shibuyunji district while the rest were done in Lusaka district. The 10 AYPLHIV who were interviewed through IDI had a lived experience with a mental health condition, hence, they were referred to as “expert clients” in this study. Of the 10 IDIs, six (6) were with males while four (4) were with female participants.

#### 2.3.2. Policy makers

We also interviewed four (4) policy makers to solicit insights on mental health policy and practice in Zambia. Of the four policy makers, one was a mental health practitioner at Zambia’s largest mental health hospital (Chainama Hills Hospital), representing the tertiary institution; one representing mental health associations/Civil Society Organization pressure group, two (2) selected from the Ministry of Health, that is, one from the provincial level (Provincial Mental Health Expert) and the other from the district level (District Mental Health Focal Point Person).

#### 2.3.3. Sampling strategy

Purposive sampling was used to ensure inclusion of diverse perspectives based on age, gender, and lived experience with mental-health challenges. AYPLHIV aged 18–24 years were identified and recruited through ART clinic registers with the support of healthcare providers and peer educators. Policymakers were identified through Ministry of Health referral pathways. Recruitment continued until thematic saturation was achieved, meaning that no new insights emerged from additional data collection.

### 2.4 Data Collection Procedures

Data was collected between February and September 2025 using semi-structured interview guides tailored for each participant group. The FGD, IDI and KII guides were developed specifically for this study, drawing on existing literature on adolescent mental health and mHealth in low- and middle-income countries, the World Health Organization service access framework, and the approved PhD protocol. Draft guides were reviewed by mental-health and HIV experts for content validity and piloted with a small group of AYPLHIV and one provider to refine wording and flow; no substantial structural changes were required following piloting. The English versions of all interview guides are provided as additional file 1.

#### 2.4.1 Focus-Group Discussions (FGDs)

Focus-group discussions (FGDs) were conducted with AYPLHIV to explore shared experiences, community perceptions, and collective challenges related to mental health and service access. A total of four FGDs were held, three in Lusaka and one in Shibuyunji, with each group comprising between 8 and 10 participants. The sessions were facilitated by trained qualitative researchers fluent in English and the predominant local languages, including Nyanja and Lenje, to ensure participant comfort and accurate expression of ideas.

#### 2.4.2 In-Depth Interviews (IDIs)

Ten in-depth interviews (IDIs) were conducted with AYPLHIV identified as “expert clients,” a group comprising youth with lived experience of mental-health challenges and established roles supporting ART adherence within their clinics. These interviews elicited detailed individual narratives, coping mechanisms, and personal encounters with the health system, providing rich insight into personal-level experiences that complemented the group perspectives gathered through FGDs.

#### 2.4.3 Key-Informant Interviews (KIIs)

Four key-informant interviews (KIIs) were conducted with policymakers representing different administrative levels, including national-level officials from the Mental Health Unit, provincial representatives from the Provincial Health office, a district Mental Health Focal Person, and a civil society representative from the Mental Health Association. These interviews explored perspectives on mental-health policy implementation, intersectoral coordination, and perceived opportunities for incorporating digital innovations within existing service structures.

All interviews and discussions were conducted in private rooms within health facilities or offices, ensuring confidentiality and comfort. Each session lasted 45–90 minutes and was audio-recorded with participants’ consent. Field notes were taken to capture contextual observations and non-verbal cues.

### 2.5 Data Management and Transcription

Audio recordings were transcribed verbatim in the original language (Nyanja or Lenje mixed with English) and all translated into English by trained transcribers experienced in health research. Quality checks were performed by the principal investigator and an independent reviewer to ensure translation accuracy and retention of meaning.

All transcripts and field notes were uploaded into Ligre software version 6.5.1 for systematic coding and data management.

To maintain confidentiality, transcripts were anonymized by removing personal identifiers and assigning participant codes (e.g., P1, IDI3, KII2). Digital files were password-protected and stored on an encrypted device accessible only to the research team.

### 2.6 Data Analysis

As this was a qualitative study, no statistical tests were conducted; instead, data were analyzed thematically using an inductive–deductive hybrid approach guided by the World Health Organization’s Service Access Framework [39] in five stages.

First, the research team undertook a period of familiarization by reading all transcripts multiple times to gain a holistic understanding of participants’ narratives. Second, initial coding was carried out by developing an inductive codebook grounded in participants’ words and lived experiences, which was then complemented with deductive codes based on the study objectives. Third, related codes were organized into broader thematic categories reflecting key patterns across participants, including understanding of mental health, availability and access to services, barriers, facilitators, and opportunities for digital integration. Fourth, themes were reviewed and refined during peer-debriefing sessions between the two primary analysts to ensure consistency, coherence, and analytic validity. Finally, the resulting themes were interpreted through the socio-ecological model of health to illustrate how individual, community, and system-level factors interact to shape access to mental-health services.

Trustworthiness and credibility were strengthened through several qualitative rigor strategies. Investigator triangulation was applied, with multiple researchers independently reviewing and comparing coded data to minimize individual bias. Data triangulation was also employed by examining and contrasting perspectives obtained from AYPLHIV and policymakers, enabling a more comprehensive and multi-layered understanding of the phenomena under study. These approaches collectively enhanced the robustness, credibility, and confirmability of the findings.

### 2.7 Ethical Considerations

#### Ethical approval

for this study was obtained from the University of Zambia Biomedical Research Ethics Committee (UNZABREC), Reference No. 5864 - 2024, and from the National Health Research Authority (NHRA), Reference No. NHRA-1679/04/11/2024. All participants provided written informed consent prior to participation. Participants were informed about the voluntary nature of the study, their right to withdraw at any time, and the measures taken to ensure confidentiality. To safeguard participants’ wellbeing, psychosocial support was made available through referral to trained counselors at participating health facilities whenever needed. All identifying details were removed from transcripts and publications.

## 3 Results

### 3.1 Participant Characteristics

A total of 54 participants took part: 40 AYPLHIV (across FGDs), 10 expert-client IDIs, and 4 policymakers. AYPLHIV were aged 18–24 years, unemployed, and attending or having completed secondary education.

### 3.2 Understanding of Mental Health

AYPLHIV conceptualized mental health as *“wellness of the mind”*, encompassing thoughts and emotions. HIV disclosure often triggered distress, anxiety, and social withdrawal:

“When you are tested positive, you become sad, with many questions like ‘Why me?’ Then you start thinking that you can’t do any work or anything productive.” (AYPLHIV, FGD)

Participants associated mental well-being with having supportive family and community relationships, while isolation and stigma led to depression and suicidal ideation.

### 3.3 Access and Availability of Services

Mental-health services were largely available through public health facilities but described as limited, irregular, and poorly resourced. AYPLHIV appreciated counseling received at ART clinics but reported that services were often unavailable or not youth-friendly:

“They are here at the facility, even in the community, but they are not based at the hospital.” (AYPLHIV, FGD)

Policymakers echoed this, noting persistent gaps in decentralization and trained personnel:

“Resources at district facilities are limited in terms of staff, infrastructure, and essential medications.” (Policymaker, KII)

### 3.4 Barriers to Access

#### 3.4.1 Socio-Cultural and Stigma-Related Barriers

Social stigma and gender norms constrained help seeking. The phrase *“Mwamuna samalila”* (“men don’t cry”) was used to describe masculine norms discouraging emotional expression:

“That saying is a challenge–society believes men cannot be vulnerable.” (Expert Client, IDI)

Cultural beliefs linked mental illness to witchcraft or curses, leading many to seek help from traditional healers instead of health facilities.

#### 3.4.2 Trust and Confidentiality

Youth expressed fear of judgment and confidentiality breaches by providers:

“We fear that what we tell them may be revealed.” (AYPLHIV, FGD)

#### 3.4.3 Economic and Geographical Barriers

Poverty and distance from health centers were major access barriers:

“Sometimes you have no money for transport, so you stay home even when you want help.” (AYPLHIV, FGD)

#### 3.4.4 Technological Barriers

High data costs and low digital literacy limited access to online counseling tools:

“Most apps use English, and people who didn’t finish school can’t understand.” (AYPLHIV, FGD)

### 3.5 Facilitators to Access

#### 3.5.1 Peer and Community Support

Peer groups and community organizations were identified as key facilitators:

“Our support group helps us talk freely; we learn it’s okay to have stress.” (AYPLHIV, FGD)

#### 3.5.2 Faith-Based and NGO Involvement

Faith-based institutions provided counseling and mental-health education. Policymakers emphasized partnerships with NGOs to expand coverage:

“Private and faith-based organizations can help fill the gap where government services are limited.” (Policymaker, KII)

#### 3.5.3 Decentralization and Provider Capacity

Policymakers stressed decentralizing mental-health services to district level and training lay counselors and peer educators:

“Training adolescents as peer providers can make services more relatable and youth friendly.” (Policymaker, KII)

### 3.6 Digital Integration

Both AYPLHIV and policymakers viewed digital tools as promising platforms for mental-health service delivery. Desired features included anonymity, data security, offline usability, and local-language options:

“The app should use different languages and have videos to help those who can’t read.” (AYPLHIV, FGD)

“Partnerships with telecom companies can make apps affordable and accessible.” (Policymaker, KII)

Youth emphasized the potential for mobile apps to reduce stigma by providing private and flexible access to counseling and psychoeducation.

## 4 Discussion

This study revealed that while AYPLHIV in Zambia understand mental health as “wellness of the mind,” access to care remains constrained by stigma, socio-cultural beliefs, economic hardship, and system inefficiencies. Five themes–understanding, access, barriers, facilitators, and digital integration–illustrate how individual experiences intersect with policy and technology contexts.

### 4.1 Understanding of Mental Health

Participants demonstrated a holistic view of mental health encompassing thoughts, emotions, and functioning, consistent with findings from other African contexts [1, 19]. Disclosure of HIV status often triggered anxiety, shame, and isolation–echoing studies in South Africa, Malawi, and Uganda where adolescents described psychological distress following diagnosis [20–22]. Limited mental-health literacy led youth to interpret symptoms as personal weakness, mirroring results from Uganda and Kenya [23, 24]. These findings underscore the need for integrating mental-health education and routine screening into ART programs to normalize care-seeking.

### 4.2 Availability and Access to Services

Both youth and policymakers described MHS as centralized and underfunded. Similar structural inequities are documented across LMICs [6, 25]. Zambia’s situation parallels WHO’s *Mental Health Atlas 2020* report that highlights severe workforce shortages and limited community outreach [26]. Counseling embedded in ART clinics offers some psychosocial support but remains inconsistent and donor-dependent–patterns reported in Kenya and Tanzania [27, 28]. Evidence from Uganda and South Africa shows that task-shifting to nurses or lay counselors effectively increases service reach [29, 30]. Implementing such models through Zambia’s Mental Health Act could decentralize services and reduce inequities.

### 4.3 Barriers to Access

#### 4.3.1 Cultural and Gender Norms

Cultural expectations, including male stoicism, discouraged emotional openness. Comparable studies in Nigeria and Uganda found that masculinized identities hinder men from acknowledging distress [31, 32]. Beliefs linking mental illness to witchcraft echo research from Ghana and Ethiopia [33, 34]. Addressing these beliefs requires community-driven education that engages traditional and faith leaders to demystify mental illness.

#### 4.3.2 Stigma and Confidentiality

Fear of “double stigma” (HIV + mental illness) limited disclosure and utilization. Similar dual stigma effects are reported across sub-Saharan Africa [7, 35]. Youth concern about confidentiality breaches mirrors Kenyan and Zimbabwean studies showing mistrust of providers [19, 28]. Training providers in ethical counseling and privacy could restore confidence.

#### 4.3.3 Economic and Geographic Constraints

Transport costs and unemployment were major obstacles, as also noted in regional research linking poverty to poor mental-health access [25, 30]. Integrating MHS into primary care and community outreach can reduce cost and distance barriers [29].

#### 4.3.4 Technological Barriers

Limited digital literacy and high data costs constrain mHealth uptake, reflecting the “digital divide” affecting rural youth across Africa [14, 17, 18]. Affordable connectivity and inclusive design are prerequisites for equitable digital-health deployment.

### 4.4 Facilitators to Access

Peer-led support, NGO involvement, and faith-based initiatives emerged as key enablers. Peer mentorship has been shown elsewhere to enhance adherence, self-efficacy, and wellbeing [20, 27]. Policymakers emphasized decentralization and staff training–approaches aligned with WHO’s *mhGAP Intervention Guide* promoting community-based task sharing [26, 29]. Public-private partnerships for service delivery and technology development mirror successful models in Kenya and India [28, 30].

### 4.5 Digital Integration

Participants viewed digital platforms as confidential spaces to overcome stigma and distance. Youth-preferred features like anonymity, multilingual content, and offline access, mirroring evidence from Uganda and South Africa where mHealth interventions improved engagement [14, 16, 27]. Policymakers’ emphasis on telecom partnerships aligns with Zambia’s *Digital Health Strategy 2022–2027* [17]. Nonetheless, sustainability depends on addressing literacy and cost barriers and involving youth in co-design, as recommended by Naslund et al [15].

### 4.6 Synthesis and Policy Implications

Findings confirm that effective improvement of youth mental-health access requires multilevel interventions. At the individual level, stigma reduction and psychosocial education are vital. At the community level, peer and faith-based networks can normalize help-seeking. At the system level, decentralization, task-shifting, and digital inclusion should be institutionalized through coordinated policy implementation. These recommendations align with WHO’s *Comprehensive Mental Health Action Plan 2013–2030* and evidence that community-anchored, technology-enabled models yield sustainable mental-health gains in LMICs [6, 26].

## 5 Study Strengths and Limitations

This study is among the first in Zambia to explore both youth and policymaker perspectives on mental-health access among adolescents and young people living with HIV (AYPLHIV).

Its methodological rigor was enhanced through triangulation of data sources (FGDs, IDIs, and KIIs), inclusion of both urban and rural settings, and use of inductive–deductive thematic analysis to ensure comprehensive theme development. The involvement of policymakers provided a systems-level understanding that complemented the lived experiences of youth.

However, several limitations must be acknowledged. First, the study was conducted in only two districts, which may limit generalizability to other regions of Zambia. Second, participants were recruited from ART clinics, potentially excluding AYPLHIV not engaged in care. Third, the sensitive nature of mental-health discussions may have led to underreporting of distress due to social desirability bias, despite efforts to ensure confidentiality. Despite these limitations, the findings provide rich, contextualized insights into barriers, facilitators, and opportunities for digital and community-based innovations in mental-health access.

As with most qualitative studies, the aim was depth and contextual understanding rather than statistical generalization; the value of these findings lies in the insights they offer to inform policy, programme design and future mixed-methods research.

## 6 Conclusion and Recommendations

### 6.1 Conclusion

This qualitative study suggests that AYPLHIV in Zambia experience substantial and layered challenges in accessing mental-health services. Participants described how stigma, gendered cultural norms, economic hardship, long distances to facilities and the centralization of specialist care combine to limit help-seeking and continuity of support. Although many AYPLHIV were aware of mental health as “wellness of the mind”, fear of judgment, concerns about confidentiality and limited youth-friendly services appeared to discourage open disclosure of distress and engagement with available services.

Policymakers in this study recognized many of the same constraints and highlighted systemic gaps, including shortages of trained providers, limited implementation of the Mental Health Act and slow progress in decentralizing services. Both AYPLHIV and policymakers perceived peer support, community involvement and carefully designed digital tools as promising ways to make services more acceptable, accessible and responsive to young people’s needs.

Given the qualitative design, purposive sampling and focus on two districts, these findings are not intended to be statistically generalizable to all AYPLHIV in Zambia. Rather, they provide rich, contextualized and transferable insights that may help inform policy, programme design and future mixed-methods research on youth mental health within HIV care.

### 6.2 Recommendations

Drawing on these findings and recognizing existing resource constraints, we suggest the following priority areas for policy, programming and research:

#### 6.2.1 Policy and System-Level Recommendations

##### 1. Progressive decentralization of mental-health services:

Policy-makers could prioritize integrating basic mental-health assessment and psychosocial counselling into primary health care and ART programmes, particularly in rural and peri-urban areas, to reduce travel and cost barriers for AYPLHIV.

##### 2. Operationalization of the Mental Health Act (2019):

Implementation of the Act may be strengthened through clearer operational guidelines, dedicated budget lines and systematic recruitment and training of mental-health professionals and lay counsellors, including those embedded in HIV clinics.

##### 3. Institutionalization of youth-friendly care:

Ministries and implementing partners could incorporate mental-health modules into adolescent HIV services, school health programmes and adolescent-friendly corners, with attention to confidentiality, respectful communication and flexible service hours.

#### 6.2.2 Community and Programmatic Recommendations

##### 4. Support for peer-led and community-based initiatives:

Formalizing peer-counsellor roles within ART clinics and providing structured supervision and training may help sustain peer-support groups that AYPLHIV already find valuable. Partnerships with NGOs and faith-based organizations could expand the reach of psychosocial support in communities.

##### 5. Stigma-reduction and mental-health literacy:

Community-level campaigns that involve traditional leaders, faith leaders and youth representatives could help reframe mental health from a purely spiritual or moral issue to one that is also social and medical, thereby normalizing help-seeking for HIV-related distress.

##### 6. Inclusive digital access and literacy:

Collaboration with telecommunications companies could be explored to reduce data costs and support zero-rating or subsidized access to approved mHealth platforms. Programmes may also invest in digital-literacy activities for young people and caregivers so that mobile applications, SMS services and hotlines are usable by those with varying education levels and device types.

#### 6.2.3 Research Recommendations

##### 7. Evaluation of hybrid mHealth and peer-support models:

Future studies could assess the feasibility, acceptability and effectiveness of combined digital and peer-support interventions for AYPLHIV across different provinces and service settings.

##### 8. Implementation and health-systems research:

Mixed-methods implementation research may help to understand how digital tools and decentralized mental-health services can be integrated into existing HIV and primary-care platforms, and how these changes influence help-seeking, adherence and retention in care.

##### 9. Participatory and co-design approaches:

Further research that actively involves AYPLHIV, caregivers and frontline providers in co-designing interventions and digital tools could ensure that proposed solutions remain culturally appropriate, acceptable and sustainable.

## 7 Policy implication summary

Addressing mental-health needs among adolescents and young people living with HIV in Zambia is likely to require coordinated action at individual, community and system levels. The findings of this study point towards the potential value of combining decentralized, youth-friendly services with strengthened peer and community support and inclusive digital innovations. If adapted to local contexts and adequately resourced, such approaches could help reduce stigma-related barriers, improve the acceptability of mental-health care and support better engagement with HIV services. While further evaluation is needed, these insights can inform ongoing efforts to implement the Mental Health Act, advance the national digital health strategy and align HIV, mental-health and adolescent-health policies in practice.

## Supplementary Material

This is a list of supplementary files associated with this preprint. Click to download.

• 1.17.2026Additionalfile1SemistructuredinterviewguidesforAYPLHIVandpolicymakersEnglishversion.pdf

## Figures and Tables

**Table 1:** Participant characteristics by interview type

Participant group	Data collection method	Number of participants
AYPLHIV	FGD	40
AYPLHIV (Expert Clients)	IDI	10
Policy Maker	KII	4
**Total**		**54**
**Source: Generated**		

## Data Availability

The datasets generated and/or analyzed during the current study are not publicly available due to the sensitivity of qualitative data and risk of deductive disclosure but are available from the corresponding author on reasonable request.

## List of acronyms

ART: Antiretroviral therapy
AYP: Adolescents and young people
AYPLHIV: Adolescents and young people living with HIV
COREQ: Consolidated criteria for reporting qualitative research
FGD: Focus-group discussion
FGDs: Focus-group discussions
HIV: Human immunodeficiency virus
IDI: In-depth interview
IDIs: In-depth interviews
KII: Key-informant interview
KIIs: Key-informant interviews
LMIC: Low- and middle-income country
LMICs: Low- and middle-income countries
MHS: Mental-health services
mHealth: Mobile health
NGO: Non-governmental organization
NGOs: Non-governmental organizations
NHRA: National Health Research Authority
SDQ: Strengths and Difficulties Questionnaire
U-Report: UNICEF’s youth digital engagement platform “U-Report”
UNZABREC: University of Zambia Biomedical Research Ethics Committee
WHO: World Health Organization
ZENITH: Zambia Education Network for Implementation Science Training in Health

## References

[1] Cluver LD, Orkin FM, Boyes ME, Sherr L. Child and adolescent mental health in HIV-affected families: A review of risk and protective factors. J Adolesc Health. 2015;57(3):331–7.

[2] Betancourt TS, Meyers-Ohki SE, Charrow A, Hansen N. Annual Research Review: Mental health and resilience in HIV-positive children and adolescents. J Child Psychol Psychiatry. 2013;54(4):423–44. 22943414 10.1111/j.1469-7610.2012.02613.x PMC3656822

[3] Collins PY, Patel V, Joestl SS, March D, Insel TR, Daar AS, Grand challenges in global mental health. Lancet. 2011;378(9803):146–57. 10.1038/475027a PMC3173804 21734685

[4] Mellins CA, Malee KM. Understanding the mental health needs of children and adolescents living with HIV: A multi-method approach. J Int AIDS Soc. 2013;16:18593. 23782478 10.7448/IAS.16.1.18593 PMC3687078

[5] Joint United Nations Programme on HIV/AIDS (UNAIDS). Global AIDS Update 2023. Geneva: UNAIDS; 2023.

[6] World Health Organization. Mental health: Strengthening our response. Geneva: WHO; 2019.

[7] Ministry of Health (Zambia). Zambia Population-Based HIV Impact Assessment (ZAMPHIA) 2021. Lusaka: Government of the Republic of Zambia; 2022.

[8] Ministry of Health (Zambia). Mental Health Act, 2019. Lusaka: Government of the Republic of Zambia; 2019.

[9] World Health Organization. Mental Health Atlas 2020: Zambia Country Profile. Geneva: WHO; 2021.

[10] World Health Organization. Mental health: Transforming access and care. Geneva: WHO; 2022.

[11] Peltzer K, Pengpid S. Mental health and help-seeking among university students in Africa: A systematic review. Afr Health Sci. 2016;16(3):820–8.

[12] Nyblade L, Stockton MA, Giger K, Bond V, Ekstrand ML, Lean RM, Stigma in health facilities: Why it matters and how we can change it. BMC Med. 2019;17:25. 30764806 10.1186/s12916-019-1256-2 PMC6376713

[13] Lund C, Brooke-Sumner C, Baingana F, Baron EC, Breuer E, Chandra P, Social determinants of mental disorders and the Sustainable Development Goals: A systematic review of reviews. Glob Health Action. 2019;12(1):1668215. 29580610 10.1016/S2215-0366(18)30060-9

[14] Donker T, Petrie K, Proudfoot J, Clarke J, Birch MR, Christensen H. Smartphones for smarter delivery of mental health programs: A systematic review. J Med Internet Res. 2013;15(10):e247. 24240579 10.2196/jmir.2791 PMC3841358

[15] Naslund JA, Aschbrenner KA, Araya R, Marsch LA, Unützer J, Patel V, Digital technology for global mental health: Opportunities for low-income and middle-income countries. Lancet Psychiatry. 2019;6(6):479–90. 10.1016/S2215-0366(17)30096-2 PMC5523650 28433615

[16] Grist R, Porter J, Stallard P. Mental health mobile apps for preadolescents and adolescents: A systematic review. J Adolesc Health. 2017;61(6):706–15. 10.2196/jmir.7332 PMC5465380 28546138

[17] Ministry of Health (Zambia). Digital Health Strategy 2022–2027. Lusaka: Government of the Republic of Zambia; 2022.

[18] UNICEF. U-Report Zambia Annual Report 2023. Lusaka: UNICEF; 2024.

[19] Mutumba M, Musiime V, Matama C, Malaba S, Kalibala S, Psychosocial challenges and coping strategies of HIV-positive adolescents in Uganda. AIDS Care. 2016;28(S2):128–35.

[20] Petersen I, Fairall L, Bhana A, Kathree T, Selohilwe O, Bhana A, Integrating mental health into chronic care in South Africa: Lessons from HIV. Health Policy Plan. 2018;33(9):1010–9.

[21] Mugisha J, Muyinda H, Wamala T, Kigozi F, Gibson L, Ssebunnya J, Stakeholder perceptions on mental health system strengthening in Uganda: A qualitative study. BMC Health Serv Res. 2020;20:992.33121477

[22] Hanlon C, Alem A, Lund C, Hailemariam D, Assefa E, Giorgis TW, Moving towards universal health coverage for mental disorders in Ethiopia. Lancet Psychiatry. 2019;6(11):935–47. 30891082 10.1186/s13033-019-0268-9 PMC6388484

[23] Mutiso V, Musyimi C, Gitonga I, Tele A, Musau A, Rebello T, Increasing mental health literacy among community health volunteers in Kenya. BMC Psychiatry. 2018;18:136.29776353

[24] Chibanda D, Mesu P, Kajawu L, Cowan F, Araya R, Abas M. Problem-solving therapy delivered by lay health workers in Zimbabwe: A cluster randomized trial. BMC Public Health. 2015;15:876.26359159

[25] Hanlon C, Fekadu A, Jordans MJD, District mental health care for low-income countries: A model from Ethiopia. Int Health. 2021;13(4):309–18.

[26] World Health Organization. mhGAP Intervention Guide (Version 2.0). Geneva: WHO; 2019.

[27] Musyimi CW, Mutiso V, Ndetei DM, Henderson DC, Bunders J. Mental health services in primary care in Kenya: A descriptive analysis. Int J Ment Health Syst. 2021;15:49.34039380

[28] Abrahams Z, Schneider M, Field S, Honikman S. Integrating digital mental health into routine maternal care: Lessons from South Africa. JMIR Ment Health. 2021;8(1):e20685.

[29] Patel V, Saxena S, Lund C, Thornicroft G, Baingana F, Bolton P, The Lancet Commission on global mental health and sustainable development. Glob Ment Health. 2018;5:e11. 10.1016/S0140-6736(18)31612-X 30314863

[30] Dube FN, Dube M, Nkomo TS. Barriers to mental health services for youth in Zimbabwe. BMC Health Serv Res. 2021;21:34.33413357

[31] Umeh C, Mbaegbu C. Masculinity norms and mental-health help-seeking among young men in Nigeria. Afr J Psychiatry. 2016;19(4):322–8.

[32] Ddamulira J, Hernandez B, Stigma, masculinity, and mental health among men in Uganda. Front Public Health. 2022;10:827551.

[33] Ae-Ngibise KA, Cooper S, Adiibokah E, Beliefs and practices regarding mental illness in rural Ghana. Int J Ment Health Syst. 2010;4:16.20565852

[34] Fekadu A, Hanlon C, Luitel NP, Mental health and illness beliefs in Ethiopia: A qualitative exploration. Lancet Psychiatry. 2019;6(11):909–20.

[35] GSMA. Mobile Internet Connectivity Report 2023. London: GSMA; 2023.

[36] Okawa S, Yasuoka J, Ishikawa N, Poudel KC, Ryu S, El-Sahn M, Psychological well-being and adherence among adolescents living with HIV in Zambia. AIDS Care. 2018;30(6):734–42. 29347827 10.1080/09540121.2018.1425364

[37] Bwalya M, Mweemba P, Choma Mental Health Study Group. Prevalence of emotional and behavioural difficulties among HIV-positive adolescents in Southern Zambia using the SDQ. BMC Public Health. 2016;16:661.27473123

[38] Tong A, Sainsbury P, Craig J. Consolidated criteria for reporting qualitative research (COREQ): A 32-item checklist for interviews and focus groups. Int J Qual Health Care. 2007;19(6):349–57. 17872937 10.1093/intqhc/mzm042

[39] World Health Organization. Monitoring the building blocks of health systems: A handbook of indicators and their measurement strategies. Geneva: WHO; 2010.

